# Difficulties in the Management of Placenta Accreta Spectrum in Hospitals with Limited Resources

**DOI:** 10.1055/s-0042-1742408

**Published:** 2022-04-26

**Authors:** Lorgio Rudy Aguilera, Luz Mariana Mojica-Palacios, Federico Urquizu, Mirko Gorena, Freddy Tinajeros Guzmán, Lina María Vergara Galliadi, Alejandra Hidalgo, Albaro José Nieto-Calvache

**Affiliations:** 1 Hospital de la Mujer Percy Boland Rodríguez, Santa Cruz de la Sierra, Bolivia; 2 Johns Hopkins University, Baltimore, MD, United States of America; 3 Centro de Investigaciones Clínicas, Fundación Valle de Lili, Cali, Colombia; 4 Clínica de Espectro de Acretismo Placentario, Fundación Valle de Lili, Cali, Colombia

**Keywords:** placenta accreta, developing countries, Latin America, placenta accreta, países em desenvolvimento, América Latina

## Abstract

**Objective** Placenta accreta spectrum (PAS) is a serious diseases, and the recommendation is that the treatment is conducted in centers of excellence. Such hospitals are not easy to find in low- and middle-income countries. We seek to describe the process of prenatal diagnosis, surgical management, and postnatal histological analysis in a low-income country referral hospital with limited resources.

**Methods** A descriptive, retrospective study was carried out including patients with a pre- or intraoperative diagnosis of PAS. The clinical results of the patients were studied as well as the results of the prenatal ultrasound and the correlation with the postnatal pathological diagnosis.

**Results** In total, 129 patients were included. Forty-eight of them had a prenatal PAS ultrasound diagnosis (37.2%). In the remaining 81 (62.8%), the diagnosis was intraoperative.

Although hysterectomy was performed in all cases, one-third of the patients (31%) did not have a histological study of the uterus. In 40% of the patients who had a histological study, PAS was not reported by the pathologist.

**Conclusion** The frequency of prenatal diagnosis and the availability of postnatal histological studies were very low in the studied population. Surgical skill, favored by a high flow of patients, is an important factor to avoid complications in settings with limited resources.

## Resumo

**Objetivo** O espectro da placenta accreta é uma patologia grave, cujo tratamento é recomendado em centros de excelência. Esses hospitais não são fáceis de encontrar em países de baixa e média renda. Procuramos descrever o processo de diagnóstico pré-natal, tratamento cirúrgico, e análise histológica pós-natal em um hospital de referência de baixa renda com recursos limitados.

**Métodos** Foi realizado um estudo descritivo, retrospectivo, incluindo pacientes com diagnóstico pré ou intraoperatório de espectro da placenta accreta. Foram estudados os resultados clínicos das pacientes, bem como os resultados da ultrassonografia pré-natal e a correlação com o diagnóstico patológico pós-natal.

**Resultados** No total, 129 pacientes foram incluídas. Quarenta e oito delas tiveram um diagnóstico de ultrassom do espectro da placenta accreta pré-natal (37,2%). Nos 81 (62,8%) restantes, o diagnóstico foi intraoperatório.

Embora a histerectomia tenha sido realizada em todos os casos, um terço deles (31%) não tinha estudo histológico do útero. Em 40% dos pacientes que tiveram estudo histológico, o espectro da placenta accreta não foi relatado pelo patologista.

**Conclusão** A frequência do diagnóstico pré-natal e a disponibilidade de estudos histológicos pós-natais foram muito baixas na população estudada. A habilidade cirúrgica, favorecida por um alto fluxo de pacientes, é um fator importante para evitar complicações em ambientes com recursos limitados.

## Introduction

Placenta accreta spectrum (PAS) is a serious disease that demands the use of many health resources, since interdisciplinary groups to complex supplies and hospital infrastructure. 1 Although it is recommended that its treatment takes place in centers of excellence, 2 such hospitals are not easy to find in low- and middle-income countries (LMICs). 3 4

The analysis of PAS fatal cases shows that, in most cases, the determining factor of death was not the lack of technological resources but the inexperience of the treating surgeons, and the use of an inappropriate surgical technique. 5 The training of the surgeon and the interdisciplinary team is probably more important than the unlimited availability of resources.

Few publications describe the clinical results of PAS management in settings with limited resources or highlight the management problems in regional referral hospitals for severe obstetric diseases in LMICs.

We seek to describe the process of prenatal diagnosis, surgical management, and postnatal histological analysis in a referral hospital for severe obstetric diseases with limited resources in a LMIC. Additionally, we aim to describe the problems identified and propose some options to overcome them.

## Methods

A descriptive and retrospective study was carried out including patients with a preoperative (by ultrasound signs) or intraoperative (applying clinical FIGO staging criteria) 6 diagnosis of PAS between January 2019 and December 2020 who were treated at Santa Cruz de la Sierra, Bolivia.

Taking into account that our hospital does not have a pathology service, and the resources available in our region require that family members be in charge of processing the histological analysis of the uterus posthysterectomy in another hospital (it is not uncommon for the report of the histological analysis to take more than 1 month or not be available to the surgical team), in all cases, a macroscopic evaluation of the surgical specimen was performed in the operating room at the end of the surgery to confirm or rule out the diagnosis of PAS. The diagnostic criteria for PAS endorsed by the International Federation of Gynecology and Obstetrics (FIGO) 6 were considered, so the patients with the following were included:

1. Histological diagnosis confirming PAS, or
2. Presence of evident clinical signs during laparotomy when observing the external surface of the uterus (purple coloring, placental bulge, and others).

The clinical results of the patients as well as the results of the prenatal ultrasound and the correlation with the postnatal pathological diagnosis were studied. Two groups of patients were established according to whether they had (group 1) or did not have (group 2) a prenatal PAS ultrasonographic diagnosis. All the patients included had placenta previa and underwent cesarean hysterectomy, applying the same operative technique ( Supplementary material 1 ). Considering that a significant percentage of patients did not benefit from the histological study of the uterus, we performed an additional analysis including only patients with histological confirmation of PAS. This retrospective study had the approval of the institutional biomedical research ethics committee - IRB/EC (N° 430-2019). A descriptive statistical analysis was carried out. Continuous variables are expressed as medians and interquartile ranges and were analyzed with the Mann-Whitney U test. The qualitative variables are summarized with absolute and relative frequencies, and the comparison between them was made with the chi-squared test or Fisher exact test, according to the case. Statistical significance was defined as *p* < 0.05. Analyses were performed using STATA version 14 software (StataCorp LP, College Station, TX, USA).

## Results

One hundred and twenty-nine patients with pre or intraoperative diagnosis of PAS were included. Table 1 describes the characteristics of the included sample. Forty-eight of them had a prenatal ultrasound diagnosis (Group 1: 37.2%), and in the remaining 81 (Group 2: 62.8%), the diagnosis of PAS was intraoperative (the prenatal ultrasound did not indicate PAS). The median maternal age was 31 years (interquartile range [IQR] 27.5–34), the median number of previous cesarean sections was two (IQR 1–3), attendance at prenatal check-ups was 63.6%, and the median gestational age at the time of surgery was 37 weeks (IQR 36–38). Although all patients had at least one prenatal ultrasound, only 41 women (31.8%) had a placental Doppler evaluation by a maternal-fetal medicine specialist. Only nine patients underwent planned surgery, and the rest underwent surgery with uterine activity or vaginal bleeding. The median volume of blood loss reported was 700 mL (IQR 500–1,000), and 8 patients (6.2% of the included cases) had bleeding equal to or greater than 2,000 mL, 7 of them in group 1.

**Table 1:** Clinical results of patients with an intraoperative placenta accreta spectrum diagnosis

		All patients (n = 129)	Group 1 patients with a presurgical diagnosis (n = 48)	Group 2 patients with intraoperative diagnosis (n = 81)	*p* -value
Maternal age (years) *		31 (27.5–34)	30 (28–34)	31 (27–35)	0.502
GA at the time of surgery (weeks) *		37 (36–38)	37 (36–37.5)	37 (36–38)	0.1794
Gravity *		3 (2–4)	3 (2–4)	3 (2–4)	0.9314
History of previous cesarean section plus placenta previa, n (%)		112 (86.8)	47 (97.9)	65 (80.2)	0.02
Number of previous cesarean sections *		2 (1–3)	2 (2–3)	2 (1–3)	0.364
Attendance at prenatal check-ups, n (%)		82 (63.6)	30 (62.5)	52 (64.2)	0.846
Performing prenatal doppler ** , n (%)		41 (31.8)	40 (83.3)	1 (1.2)	0
Planned surgery, n (%)		9 (7)	7 (14.6)	2 (2.5)	0.013
Intraoperative bleeding volume *		700 (500–1,000)	700 (500–1,000)	700 (500–900)	0.3282
Bleeding volume ≥ 2,000 ml, n (%)		8 (6.2)	7 (14.6)	1 (1.2)	0.004
RBCU transfusion frequency, n (%)		102 (79.1)	41 (85.4)	61 (75.3)	0.173
RBCUs transfused *		2 (1–2)	2 (1–2)	2 (1–2)	0.4795
Transfusion ≥ 4 RBCUs, n (%)		9 (7)	5 (10.4)	4 (4.9)	0.048
Presurgical Hb (gr/dL) *		11 (9.6–11.6)	11 (9.8–12.1)	10.6 (9.4–11.4)	0.0516
Postsurgical Hb (gr/dL) *		10 (8.9–11.3)	10.65 (8.9–11.4)	10 (8.8–11)	0.2932
Conductive anesthesia (spinal or epidural), n (%)		125 (96.9)	47 (97.9)	78 (96.3)	0.893
Surgery between 7 pm and 7 am, n (%) ***		48 (47.1)	17 (43.6)	31 (49.2)	0.723
Accreta (clinical diagnosis), n (%)		106 (82.2)	34 (70.8)	72 (88.9)	0.005
Increta-Percreta (clinical diagnosis), n (%)		22 (17.1)	14 (29.2)	8 (9.9)	0.005
Result of the histological study, n (%)	No histological study	40 (31)	15 (31.5)	25 (30.9)	0.23
	Normal	35 (27.1)	8 (16.7)	27 (33.3)	0.012
	Accreta	40 (31)	17 (35.4)	23 (28.4)	0.32
	Increta and Percreta	14 (10.8)	8 (16.7)	6 (7.4)	0.03
Admission to ICU, n (%)		17 (13.2)	9 (18.8)	8 (9.9)	0.15
Days of postoperative hospitalization *		4 (3–4)	4 (3–4.5)	4 (3–4)	0.4818
Ureteral injury, n (%)		2 (1.6)	1 (2.1)	1 (1.2)	1
Bladder injury, n (%)		8 (6.2)	4 (8.3)	4 (4.9)	0.469
Surgical reintervention, n (%)		4 (3.1)	2 (4.2)	2 (2.5)	0.628

Abbreviations: GA, Gestational age; ICU, intensive care unit; PAS, placenta accreta spectrum; RBCU, red blood cell unit

* Median (Interquartile range -IQR-)

** By a maternal fetal medicine (MFM) specialist

*** Calculations among 102 cases with information (39 with prenatal diagnosis, 63 without prenatal diagnosis)

One hundred and two patients (79.1%) received red blood cell transfusion, with a median of two red blood cell units (RBCUs) (IQR 1–2). Nine patients (7%) received four or more RBCUs. The median preoperative hemoglobin was 11 g/L, and the last hemoglobin before discharge was 10 g/L (median).

Neuraxial anesthesia was used in 96.9% of the patients (125 women), and 47.1% of the surgeries were performed at night (between 7:00 p.m. and 7:00 a.m.). Although all the patients were managed with a hysterectomy and had intraoperative confirmation of PAS when a cut of the operative piece was performed by the main surgeon, a third of them (31%) did not have a histological study of the uterus. In 40% of the patients who had a histological study (27% of the population), PAS was not reported by the pathologist. The intraoperative clinical diagnosis was accreta in 106 patients (82.2%) and increta/percreta in 22 patients (17.1%). The frequencies of admission to the intensive care unit, ureteral injury, bladder injury, and surgical reintervention were 13.2%, 1.6%, 6.2%, and 3.1%, respectively. The median length of postoperative hospitalization was 4 days (IQR 3-4). Group 1 had a higher frequency of previous risk factors for PAS (previous cesarean section plus placenta previa), placental Doppler evaluation, and planned surgery. Group 1 also had a higher frequency of placenta increta/percreta in the histological study, bleeding greater than 2,000 mL, and transfusion of more than four RBCUs. When analyzing only the patients with histological PAS confirmation (n = 54, 41.9%), a higher frequency of more than two liters of bleeding and more than 4 RBCU transfusions persisted in the group with prenatal diagnosis ( Table 2 ), with no other differences from what was observed in the general population. No maternal deaths were observed.

**Table 2:** Clinical results of patients with a histological confirmation of placenta accreta spectrum

	**All patients (n = 54)**	**Group 1** **patients with a presurgical diagnosis (n = 25)**	**Group 2** **patients with intraoperative diagnosis (n = 29)**	***p*** **-value (comparing groups 1 and 2)**
Maternal age (years) *	31 (28–34)	31 (28–34)	32 (28–37)	0.2202
GA at the time of surgery (weeks) *	37 (35–38)	37 (35–37)	37 (35–38)	0.4695
Gravity *	3 (3–4)	3 (3–4)	3 (3–4)	0.6942
History of previous cesarean section plus placenta previa, n (%)	50 (92.6)	25 (100)	25 (86.2)	0.115
Number of previous cesarean sections *	2 (2–3)	2 (2–3)	2 (1–3)	0.5255
Attendance at prenatal check-ups, n (%)	29 (53.7)	12 (48)	17 (58.6)	0.435
Performing prenatal doppler ** , n (%)	22 (40.7)	22 (88)	0 (0)	0
Planned surgery, n (%)	5 (9.3)	3 (12)	2 (6.9)	0.653
Intraoperative bleeding volume *	700 (500–1,750)	700 (550–1,750)	800 (500–1,000)	0.559
Bleeding volume ≥ 2,000 ml, n (%)	7 (13)	6 (24)	1 (3.4)	0.038
RBCU transfusion frequency, n (%)	44 (81.5)	23 (92)	21 (72.4)	0.086
RBCUs transfused *	2 (1–3)	2 (1–3)	2 (1–3)	0.4972
Transfusion ≥ 4 RBCUs, n (%)	4 (7.4)	3 (12)	1 (3.4)	0.034
Presurgical Hb (gr/dL) *	11 (9.6–12)	11.6 (10.5–12.5)	10.7 (9.5–11.5)	0.0396
Postsurgical Hb (gr/dL) *	9.95 (8.4–11)	10.8 (8.9–11.4)	9.35 (8.35–10.7)	0.1318
Conductive anesthesia (spinal or epidural), n (%)	43 (98.1)	25 (100)	28 (96.6)	1
Surgery between 7 pm and 7 am, n (%) ***	13 (24.1)	5 (20)	8 (27.6)	0.745
Accreta (clinical diagnosis), n (%)	40 (71.1)	17 (68)	23 (79.3)	0.017
Increta-Percreta (clinical diagnosis), n (%)	14 (25.9)	8 (32)	6 (20.68)	0.042
Admission to ICU, n (%)	12 (22.2)	8 (32)	4 (13.8)	0.109
Days of postoperative hospitalization *	4 (3–6)	4 (3–7)	4 (3–5)	0.3751
Ureteral injury, n (%)	2 (3.7)	1 (4)	1 (3.4)	1
Bladder injury, n (%)	6 (11.1)	4 (16)	2 (6.9)	0.399
Surgical reintervention, n (%)	3 (5.6)	2 (8)	1 (3.4)	0.591

Abbreviations: GA, gestational age; ICU, intensive care unit; PAS, placenta accreta spectrum; RBCU, red blood cell unit

* Median (Interquartile range -IQR-)

** By a maternal fetal medicine (MFM) specialist

*** Calculations among 102 cases with information (39 with prenatal diagnosis, 63 without prenatal diagnosis)

## Discussion





The reality of PAS management in the studied sample is different from the model described in centers of excellence in PAS. The performance of the prenatal diagnosis for PAS and the efficacy of the postnatal pathological study in one of the hospitals with the highest number of births per year in an LMIC are very low.

Despite limitations in prenatal diagnosis, the frequency of complications and the need for transfusion of more than four RBCUs were low.

Multiple publications recommend the treatment of patients with PAS in specialized hospitals with trained interdisciplinary groups. 1 2 However, the characteristics of these “centers of excellence for PAS” are fulfilled by very few hospitals in LMICs 3 4 and even in developed countries. 7 Although the establishment of demanding quality standards favors the improvement of processes in hospitals, it is essential to measure the real baseline situation in each center. Most Latin American hospitals treat a patient with PAS every 2 months, as only 11 centers were identified in the region with more than 2 cases per month attended by a “PAS team”. 4 Our observations regarding the low performance of prenatal ultrasonographic diagnosis for detecting PAS and the frequent absence of postoperative histological studies are likely shared by other hospitals in LMICs. 8 9

Some results of this study differ from observations in other populations and allow one to suspect inaccuracy in medical records. Such low blood loss volumes may be due to an underestimation of bleeding because no objective methods were used to quantify blood loss in our hospital; only visual estimation was employed. The inclusion of patients with placenta previa and without accreta was not ruled out. However, if we limited ourselves to analyzing only the 54 patients with histological confirmation of PAS ( Table 2 ), our center would report 2.2 cases per month. This high frequency of patients managed by the same medical group leads us to believe that the surgical experience of the treating group is the reason for the low frequency of complications observed, despite the limited resources available ( Tables 1 and 2 ). The frequency of bladder injuries of 11% ( Table 2 ) is much lower than the 27% reported in other Latin American countries 10 or some centers of excellence for PAS. 11 Something similar happened with the observed frequency of transfusions of more than 4 RBCUs (7%), which is low when compared with other series, where up to 25% of the population required that number of RBCUs. 11

Some results observed are due to the surgical protocol used and not necessarily the severity of the pathology. The high frequency of transfusions (79.1%) with hemoglobin at a discharge of 10 g/L may suggest that some of these transfusions were not necessary and were due to the “excessive” caution of the treating team. The management protocol in our center considers the nonavailability of additional strategies to control bleeding (interventional radiology, cell saver, support from specialists in vascular surgery) and allows a low threshold for transfusion ( Supplementary material 1 ).

This study is the first formal analysis of our results and allows us to observe several opportunities for improvement involving an intervention plan that must be coupled with the “mother-child” nature of our hospital ( Chart 1 ).

**Chart 1:** Improvement opportunities for the care of placenta accreta spectrum patients in a low- and middle-income country maternal and child hospital

Identified improvement option		Possible solutions
Low prenatal diagnostic performance of PAS	Insufficient knowledge of the disease by prenatal control personnel. Nonreferral of women with RF.	Screening for PAS in high-risk populations
	Insufficient knowledge of the disease by obstetricians who perform basic ultrasonography. No active search in population with RF.	Periodic webinars about PAS Virtual continuous support to sonographers by MFM specialists
	Insufficient availability of MFM specialists. Not timely access to specialized evaluation of patients with RF.	Periodic education for professionals Patients with PAS RF prioritization for access to MFM specialist
End of pregnancy with more than 36 weeks of gestation	Insufficient availability of neonatal ICU	Administrative and economic efforts to expand the number of neonatal ICU beds Schedule in advance the birth of these babies to have on notice to the neonatal ICU Visibility of PAS and search for private companies or government support, seeking greater availability of ICU beds (economic support)
	Insufficient opportunity for an operating room due to the high number of daily cesarean section births	Recognition of the risk of severe maternal and neonatal morbidity in late emergent surgery Schedule in advance these surgeries to have "on notice" to the operation room
	Late catchment of the patients	PAS RF patients active search by a specialized center Early identification of the disease
Lack of an interdisciplinary group	The “mother-child” nature of the hospital	Assessment of the adequacy of this hospital model Support from nearby hospitals in scheduled surgeries Remote support of interdisciplinary groups to adapt the protocol to the available personnel
	Difficulties for agile hiring of additional personnel for specific procedures	Administrative effort focused on facilitating the necessary hiring
	Lack of collaborative work with neighboring private hospitals	Creation of regional groups for academic discussion and assistance support around PAS
	Absence of a “PAS team”	Identification of leaders interested in PAS in each specialty (anesthesiology, pediatrics, obstetrics, intensive care, nursing, surgical instrumentation, etc.)
	Lack of contact with groups in other cities or countries	Use of telemedicine and participation in regional (LatAm PAS study group) and international (IS-PAS, PAS2) academic groups
Absence of feedback on histological study results	Insufficient availability of pathology services in the region linked to the public health system	Administrative effort focused on facilitating the necessary hiring
	Absence of pathology service within the hospital	Administrative effort focused on facilitating the necessary hiring
	Lack of institutional messaging system to transfer surgical piece to pathology service	Administrative effort focused on facilitating the safe remission of surgical pieces
	Lack of communication with pathologists from another institution	Inclusion of local or regional pathologists in interdisciplinary groups
	High frequency of normal reports of cases with apparent PAS in the macroscopic examination during surgery. It may be related to the fact that surgeons always detach the placenta after surgery to confirm the presence of PAS in the face of the low frequency of histological studies.	Avoid placental delivery in the operating room by surgical group, provided that histological processing is ensured to clarify the diagnosis.
Limited capacity for massive transfusion	Absence of a blood bank inside the hospital	Administrative effort focused on facilitating the necessary hiring
	Variable response to the emerging request for blood components other than RBCU	Administrative effort focused on facilitating the agile and sufficient supply of blood components from external blood banks
	Absence of intraoperative cell recovery system (“cell saver”)	Hospital economic investment for the acquisition of the equipment Request to nongovernmental organizations to donate this equipment (for example Jehovah's witnesses)
High frequency of “emergent” surgeries (with vaginal bleeding or uterine activity) or at night	Surgeries scheduling between 34–36 weeks	Construction, disclosure, and supervision of compliance with institutional protocol for PAS
	Lack of prioritization of patients with PAS RF (previous cesarean section and placenta previa) in surgical programs	Recognition of the risk for severe maternal and neonatal morbidity in late emergent surgery

Abbreviations: ICU, intensive care unit; MFM, maternal-fetal medicine; PAS, placenta accreta spectrum; RBCU, red blood cell unit; RF, risk factors

Although the model of centers of excellence for PAS requires the permanent availability of a large number of specialists, 12 our hospital attends the majority of births in the largest state of Bolivia, 13 and there is no other nearby public hospital with better resources to attend PAS patients. It is clear that patients with PAS will continue to be cared for in our center. It is, therefore, necessary to plan medium- and long-term interventions to improve the quality of care in our center ( Chart 1 ).

This study has several limitations that must be taken into account to analyze the results. Its retrospective nature allows bias to occur, and there is a possibility that some data collected in hospital records may not be accurate.

The absence of histological analysis in 31% of the cases, and normal histology results in another 27.1% of the included patients represent a problem for the analysis of our results, as it is likely that some of the patients did not have PAS.

Also, the high frecuency of PAS clinical diagnosis without histological correlation, reflects the importance of including pathologist among the PAS interdisciplinary groups and the dificulties for carrying out high quality research in LIMCs. We performed an additional analysis including only patients with histological confirmation of the PAS clinical diagnosis ( Table 2 ), and the results are very similar to those observed in Table 1 .

This paper intends to show the reality of PAS management in one of the largest maternal hospitals in an LMIC. Although the majority of publications in indexed journals show successful experiences, and it is difficult for hospitals with limited resources to develop quality research activities, the reality in our center is the same as that of a significant percentage of institutions that attend PAS patients, at least in LMICs. Commonly, surgery for PAS and histological study of the uterus are carried out in different hospitals, and surgeons do not know the opinion of the pathologist (and vice versa).

In our center, the surgeon always performs a macroscopic analysis of the surgical specimen ( videos 1 and 2 ). It is likely that detachment of the placenta from the uterus in the operating room (after surgery) limits the histological observation of superficial degrees of invasion, and that this explains at least in part the low correlation between the intraoperative diagnosis and the histological diagnosis. Additionally, there was no communication between the surgical group and the pathologists, which has been shown to deteriorate the performance of histological diagnosis. 14

When we compare our practice to the recommendations of the centers of excellence, most of the opportunities for improvement identified in our management protocol ( Chart 1 ) make it clear that the management of PAS is not optimal in our center. However, it is important to recognize the difficulties of PAS management in LMICs. In some regions, there is simply no other better-equipped hospital to refer PAS patients to.

Most likely, our observations are not exact, but we do not doubt that our results are at least an approximation of the reality in our country.

Among the options to bring the two realities closer (that of the centers of excellence for PAS and that of the Latin American “maternal and child” hospitals) are interinstitutional collaboration by telemedicine, and the incorporation of research into healthcare practice. 3 15 These two activities require the specific training of the participants, incorporation of the best available scientific evidence, greater vigilance of care processes, and fluid contact with other groups dedicated to the management of PAS. Although an economic investment and a great administrative effort are necessary, the availability of obstetricians with extensive exposure to PAS is a favorable factor. In addition, bringing public interest to the problems of managing this disease through scientific publications facilitates investment from public health entities.

Many countries have a reality that is similar to ours in the management of PAS, with few or no hospitals with all the characteristics described for a PAS center of excellence. The first step on the path of change is to expose this situation. Multicenter prospective studies are necessary for Latin America to evaluate the real situation of care for women with PAS, as well as the formation of international academic networks that support the management of this disease in our region.

## Conclusion

The frequency of prenatal diagnosis and the availability of postnatal histological studies were very low in the studied sample. Surgical skill, favored by a high flow of patients, is an important factor to avoid complications in settings with limited resources.
