# Supplementary material for: Difficulties in the Management of Placenta Accreta Spectrum in Hospitals with Limited Resources
Source: Rev Bras Ginecol Obstet. 2022 Apr 26;44(5):467–74. doi: 10.1055/s-0042-1742408 (PMC9948092; doi:10.1055/s-0042-1742408)
Supplement: Supplementary file 3 — Supplementary Material [file 10-1055-s-0042-1742408-s210385.pdf]

Supplementary material 1

Surgical technique for placenta accreta spectrum (PAS) used at the Dr. Percy Boland Rodríguez Women's Hospital in Santa Cruz de la Sierra, Bolivia.

Identification of all patients with risk factors for PAS (history of cesarean section and placenta previa)

Evaluation of prenatal images

Knowledge of the low performance of ultrasound, all patients with PAS risk factors receive median infraumbilical laparotomy

Two peripheral venous access numbers 14-18

Nonpneumatic anti-shock suit available

2-3 red blood cell units (RBCUs) reserve

Neuraxial anesthesia (spinal or epidural)

Median infraumbilical skin incision

Visual confirmation of PAS (FIGO criteria)

Enlargement of the skin incision if PAS is confirmed

Externalization of the gravid uterus

Transverse fundic hysterotomy

Fetus extraction leaving placenta in situ

One plane hysterorraphy

Total hysterectomy

Clamping, cutting, and ligation of the round ligament

Clamping, cutting, and ligation of the uterus-ovarian ligament

Slow and meticulous dissection of the vesicouterine space

Suture ligation of vessels that run between the uterus and bladder

Skeletonization and ligation of uterine arteries

Identification and lateralization of the ureter

Clamping, cutting, and ligation of the broad ligament to the most caudal part

Vaginal dome cut and closure

Removal of the surgical piece (uterus)

Hemostasis check with clamping and ligation of bleeding vessels on the posterior aspect of the bladder and lateral walls of the pelvis

In abnormal bleeding was observed or massive bleeding was predicted, a "low threshold" was used for RBCU transfusion. This was motivated by the absence of additional strategies to control bleeding (interventional radiology, specialists in vascular surgery) or the replacement of massive blood losses (cell saver, blood bank inside the hospital) in our hospital.

Administration of tranexamic acid when intraoperative bleeding exceeds 1000 mL

Parietal peritoneum closure

Abdominal wall closure

Postoperative management depending on blood loss (recovery room, obstetric unit, or intensive care unit)

 © 2022. Federação Brasileira de Ginecologia e Obstetrícia. All rights reserved.
